# Metal-Organic Frameworks as advanced moisture sorbents for energy-efficient high temperature cooling

**DOI:** 10.1038/s41598-018-33704-4

**Published:** 2018-10-16

**Authors:** Shuqing Cui, Menghao Qin, Afsaneh Marandi, Victoria Steggles, Sujing Wang, Xiaoxiao Feng, Farid Nouar, Christian Serre

**Affiliations:** 1 Department of Civil Engineering, Technical University of Denmark, Lyngby, 2800 Denmark; 2 Institut des Matériaux Poreux de Paris, FRE 2000 CNRS, Ecole Normale Supérieure, Ecole Supérieure de Physique et de Chimie Industrielles de Paris, PSL Research University, 75005 Paris, France; 3 Elektron Gri, 75005 Paris, France

## Abstract

Latent cooling load accounts for 30% of the total load of air-conditioning, and its proportion is even higher in many tropical and subtropical climates. Traditional vapour-compression air-conditioning (VCAC) has a low coefficient of performance (COP) due to the refrigeration dehumidification process, which often makes necessary a great deal of subsequent re-heating. Technologies using conventional desiccants or sorbents for indoor moisture control are even less competitive than VCAC due to their high regeneration temperature, long cycling time and bulky components. Here, we report a novel high temperature cooling system that uses porous metal-organic frameworks (MOFs) as advanced sorbents for humidity control. We directly coat MOFs on the surface of evaporator and condenser. The system has no additional components compared to a traditional VCAC. The evaporator can simultaneously remove both the sensible and latent loads of the incoming air without reducing the temperature below its dew point. The regeneration of wet MOFs is completely driven by the residual heat from the condenser. The MOF-coated heat exchangers can achieve a cooling power density of 82 W·L^−1^. We demonstrate that the system has a high COP, up to 7.9, and can save 36.1% of the energy required, compared to the traditional VCAC system with reheating. The amphiphilic MOFs used in the research have high water uptake, are made of low-cost raw materials and have high hydrothermal stability. They thus have the potential for being scaled up for large-scale applications in air conditioning.

## Introduction

Buildings are responsible for 30% of global annual greenhouse gas emissions and consume up to 40% of all energy. Energy demand in the building sector could increase by another 30% in the next forty years if no further actions are taken^1^. Transitions to the 2 °C targets set in the Paris Agreement require the rate of increase to be reduced by 50%. Outpacing other end uses in buildings, the growth in cooling energy demand has multiplied by 2 and 5 times respectively in OECD and non-OECD countries since 1990^1^. There is an urgent need to develop energy-efficient cooling technologies to reduce building energy consumption. Conventional vapour-compression air conditioners normally have a coefficient of performance (COP) around 3 in practice^2^. The low COP is primarily due to the refrigeration dehumidification process. By cooling the air below the dew point, the latent load (humidity load) is removed by condensation. A great deal of subsequent re-heating is required^3^ to increase the air temperature to meet the supply-air requirement for indoor thermal comfort^4^. Typically, the latent part accounts for around 30–40% of the working load in air-conditioning, and its proportion is even higher in some hot and humid climates^5^. Considerable efforts have been made to develop alternative air-conditioning technologies^6–8^. High temperature cooling made possible by novel sorbent or desiccant materials is a promising approach^9^, where a sorption and heat-driven desorption process handles the removal of moisture. Dealing only with the sensible load, the system can raise the evaporation temperature from a typical 5–7 °C to a higher range (e.g., 15–20 °C^10^) so the COP and energy efficiency of the system can be dramatically improved^11,12^.

In principle, sorbent or desiccant materials can be divided into two categories: liquid and solid. Liquid sorbents are mainly based on a solution of hygroscopic salts. Dehumidification by liquid sorbents uses less electrical energy than refrigeration, but the relevant technology has distinct drawbacks for commercialization, e.g. it requires a complex system and has corrosion problems^13^. Subject to low water uptake capacity and requiring a high regenerative temperature, conventional solid sorbent systems using silica gel or aluminium-rich zeolites, though less bulky than that of liquid sorbent, are restrained by the availability of heat sources^14^. Some studies have suggested combining liquid and solid sorbents, for example, by encapsulating different salts into a porous matrix^15^. The water uptake capacity of the mixture can be enhanced, but corrosion is still problematic, especially when the composite sorbents are directly applied to a metal surface^16^.

Metal-organic frameworks (MOFs) are a class of porous crystalline materials that consist of metal clusters and organic linkers. Due to their high porosity and large specific surface, MOFs can be used for gas storage, purification and catalysis, etc. Recent studies show that MOFs are also promising sorbents for water vapour. The diverse choice of linkers and secondary building blocks (SBU) facilitate the modulation of the hydrophilicity and sorption kinetics^17–22^. Some of us have reported carboxylate-based MOFs showing remarkable uptake for water and requiring lower regeneration temperatures for the desorption compared to conventional sorbents. We found that these MOFs would be excellent sorbents for moisture control in the built environment due to their high hydrothermal stability, non-toxicity and non-corrosion^23^.

**Figure 1:**
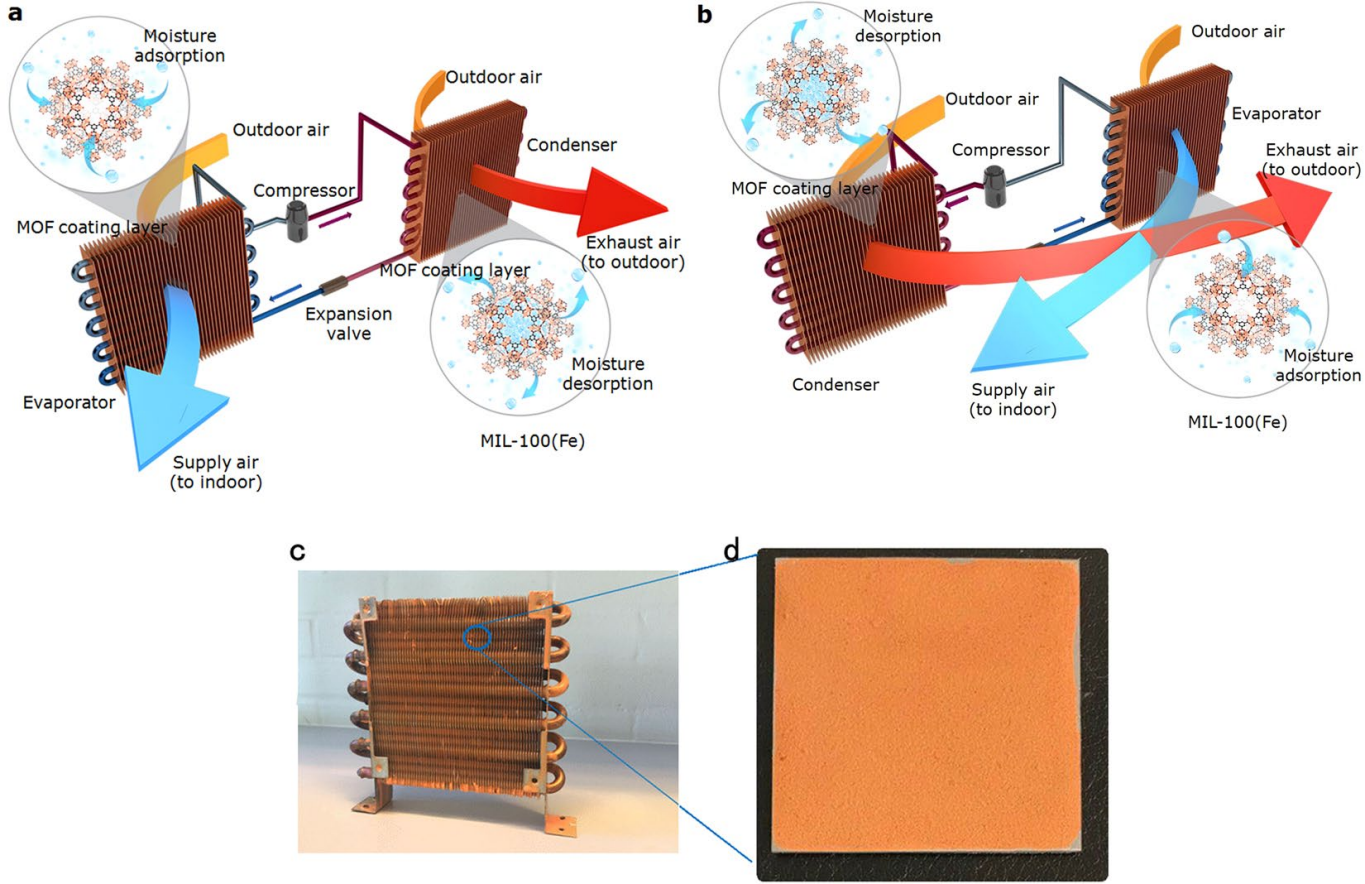
Schematic illustration of the working mechanism of a MOF high temperature cooling system. (**a**) MOFs absorb, store and transfer latent heat loads. The MOF HEx on the left side is in the adsorption mode and works as an evaporator. The MOF HEx on the right side is in the desorption mode and works as a condenser. (**b**) When the MOF coating on the evaporator is saturated, the direction of refrigerant circulation will be reversed, the MOF HEx on the left side becomes a condenser and that on the right side becomes an evaporator. (**c**) Image of a MOF HEx coated with MIL-100(Fe). (**d**) Image of a MOF HEx Element: a single aluminium fin with single-side-coated MIL-100(Fe).

In this paper, we report a new high temperature cooling system integrated with MOF-coated heat exchangers. The system has no additional components compared to a traditional VCAC (Fig. 1). We directly coated the MOFs on the surface of a metallic heat exchanger (HEx) that works in two modes. In the adsorption mode, the MOF HEx works as an evaporator, maintaininga low temperature that is slightly higher than the dew point of the incoming air. The hot and humid outdoor air passes through the MOF HEx and is dehumidified and cooled to the conditions required for the supply air. In this process, both sensible and latent loadsare removed simultaneously without moisture condensation. In the desorption mode, the MOF HEx acts as a condenser when the sorbent is saturated. The regeneration of the wet MOF coating is completely driven by the heat from condensation of refrigerant. No additional energy is needed. The exhaust air from the condenser is directly discharged to the outdoor environment. Two identical MOF HEx units can ensure the continuous operation of the system by simply reversing the direction of refrigerant and air flow between the two modes.

We selected those amphiphilic MOFs primarily exhibiting a high water-uptake in the pressure range of 25–50% relative humidity (RH). Hydrophilic MOFs often shows the inflection points of water isotherms at a low relative humidity (<25%) and a relatively high sorption enthalpy due to the strong interaction between water molecules and the functional groups/SBUs^24,25^. Overly hydrophobic MOFs, in contrast, are unable to dry the incoming air to the required level of supply air for the building^26,27^.

**Figure 2:**
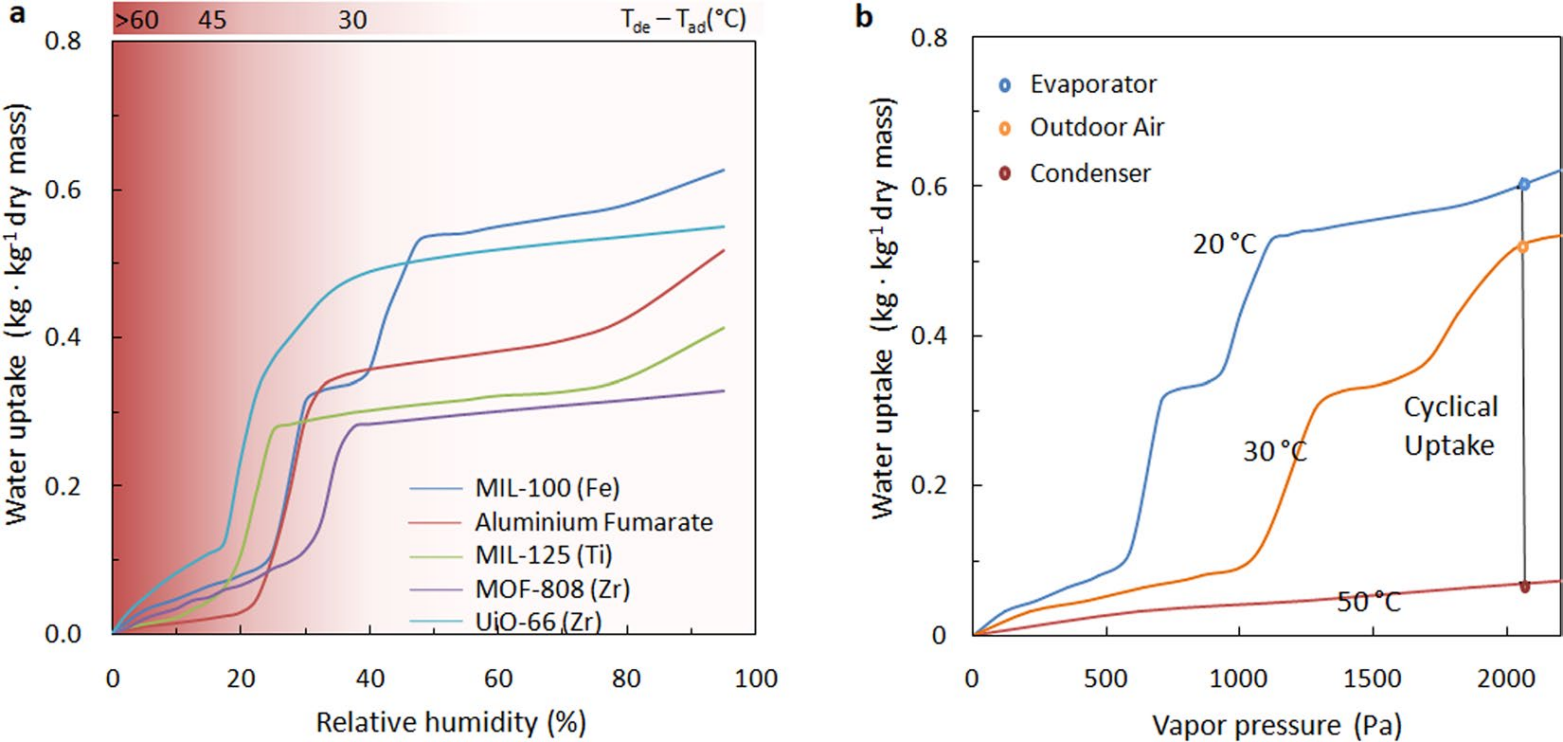
(**a**) Water adsorption isotherms of typical amphiphilic MOFs with simple linkers (MIL-100(Fe), Basolite A520 (Aluminium Fumarate), MIL-125(Ti), UiO-66(Zr), MOF-808(Zr)) at 25 °C, with the steep step located in the narrow range of RH 25–50%. The operation window of a cooling system is illustrated by the difference between the temperatures of the ambient air and the dew point (SI5, T_evaporator_ > T_dew point_). The more hydrophilic the materials are, the more significant the temperature lift that can be achieved (T_ambient_ − T_evaporator_), while a higher temperature of desorption is required. (**b**) Temperature swing operation with MIL-100(Fe). Water adsorption isotherms measured at 20 °C, 30 °C and 50 °C show that the cyclical loading difference is 0.56 kg·kg^−1^ dry mass at 2100 Pa vapour pressure (50% RH at 30 °C).

The delicate choices on these MOFs allow the system to maximally exploit the unique S-shape isotherms of the MOFs so that the cyclical water uptake is close to the maximum adsorption of the dry materials. The system can operate efficiently even though the regeneration is powered by a very low-grade heat source (Fig. 2). With the present concept, the waste heat from the condenser with a temperature lower than 50 °C can be used for moisture desorption. We estimated that the present system can reach a high COP of 7.9 on a typical summer day in an oceanic climate (e.g. Europe) while maintaining high specific cooling power (SCP).

### Construction of the MOF Heat Exchanger

In addition to the steep step in the suitable RH range on the isotherms, the sorbent must possess other advantages such as high water-uptake, low-cost, a lack of toxicity and the possibility of scalable production. High-valent metals (III, IV) carboxylates, including hierarchical mesoporous or microporous structures built with the most uncomplicated aromatic rings, e.g., terephthalate/trimesate, and fumarate^28–33^, are good candidates to meet all these requirements. The organic linkers without functionalization can be directly purchased on commercial markets, and synthesis can be achieved by robust and straightforward routes.

Many types of MOFs have been synthesized in the past several years^34^, but few of them meet the above criteria. We have investigated the performance of a few archetypal amphiphilic MOFs, including MIL-100(Fe), Basolite A520 (Aluminium Fumarate), MIL-125(Ti), UiO-66(Zr), and MOF-808(Zr), etc. With one of the highest water uptake capacity ever reported, MIL-100(Fe) has a better overall performance than any other. We therefore chose MIL-100(Fe) for use in a demonstration of the system concept. The crystalline structure of MIL-100(Fe) has a rigid three-dimensional cubic form, composed of oxo-centred iron(III) octahedral trimers linked to trimesate ligands [Fe_3_O(H_2_O)_2_(OH)(BTC)_2_], creating two mesoporous cavities of 25 and 29 Å. The heat released from the exothermic adsorption within our operation is very close to the latent heat^23^. The synthesis of MIL-100(Fe) was at the scale of several hundreds of grams in a batch at the laboratory level and can easily be increased to continuous production with high space-time yields^35^ (Supplementary Information SI1).

To overcome the inherent problems of weak mechanical strength and low density^36^, we shaped the activated MIL-100(Fe) powders into macroscopic layers on a metal surface. The concept of coating also allows the MOFs to rapidly dissipate or absorb heat under isothermal conditions via the evaporator or condenser, which guarantee the desired sorption performance. The coating process used a water-borne binder of silica sol (an aqueous silicic acid solution with water-insoluble silicon dioxide in colloid distribution), enabling the MOF layer to retain its original water sorption features and capacity (besides the “dead volume” on the silicates). The characteristic behaviour of filling the mesopores in two consecutive steps observed in MIL-100(Fe) crystalline material^37^ was well conserved. The optimized recipe showed both hydrothermal and mechanical stability after three months of sorption cycle tests without peeling off (SI2).

### Water sorption characterization

**Figure 3:**
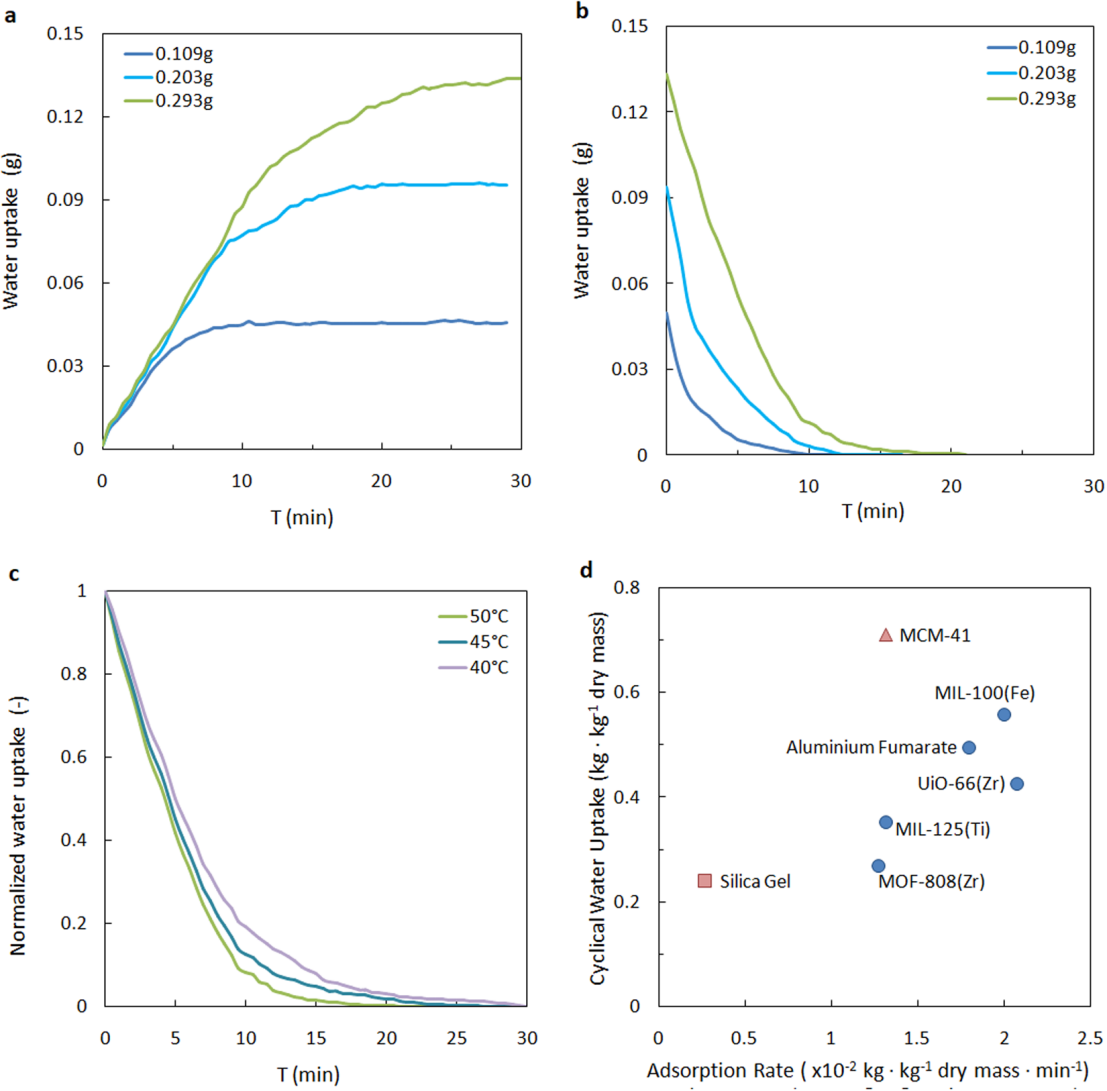
Water sorption dynamic achieved by the MOF Hex Element. (**a**) Adsorption curves for three coated MIL-100(Fe) layers of 0.109 g, 0.203 g, and 0.293 g at 20 °C. (**b**) Desorption curves for three coated MIL-100(Fe) layers of 0.109 g, 0.203 g, and 0.293 g at 50 °C. (**c**) Normalized desorption curve for coated MIL-100 layer of 0.293 g at 50, 45 and 40 °C. (**d**) Water cyclical load capacity (20–50 °C) and adsorption rates of common sorbents. In the mass calculation of the MOFs the binder weight was subtracted. The adsorption rates are averaged values for 0–90% water loading with coated layers of 450 µm thick.

We investigated the ability of a representative element of the coated MOF HEx to perform the macroscopic ad-/desorption characterization (Fig. 3). Three samples of MIL-100(Fe) were coated on one side of 4 cm × 4 cm × 0.03 cm aluminium plates, obtaining a mass of 0.109 g, 0.203 g, and 0.293 g (90 wt% MOF and 10 wt% binder) and a thickness of 157 ± 37 µm, 313 ± 55 µm, and 463 ± 64 µm. The reverse side of the aluminium plate was attached to a Peltier cooler (TEC) to control the surface temperature, then the MOF Hex Element was placed into a small climate chamber with controlled temperature and RH.

The principle of the characterization experiments was the same as under real-life conditions. We provided an isobaric environment with the small climate chamber, with reference to the ambient atmosphere, and represented the evaporator/condenser parts with the TEC (SI3). To represent a typical summer day in Europe, the chamber was set to 30 °C, RH 50%. In the adsorption mode, the surface temperature of the HEx Element was controlled to be 20 °C. The total amount of water that the coated layer could adsorb was 0.45 kg·kg^−1^ dry mass, estimated from the equilibrium states on the isotherms and was measured instantaneously using a gravimetric method. In the desorption mode, the current passing through the TEC was reversed, and the surface temperature of the HEx Element increased to 50 °C. The time-dependent sorption curve validated the tight relation between the layer thickness and the dynamics. The three coating layers reached 90% saturation in 7, 13 and 18 minutes, respectively (Fig. 3a).

We can calculate the energy efficiency of a sorption cycle in which the MOF layer operated as the latent heat storage and transfer media^38^. MIL-100(Fe) has a reversible adsorption/desorption cycle, and the effect of hysteresis within the working range is negligible. The latent load removed is equal to the thermal energy input minus the heat loss, which is mainly due to the parasitic sensible loads during the mode switch between evaporator and condenser. A thinner coating layer implies better heat transfer performance, which is essential to ensure quasi-isothermal sorption of the moisture. However, an augmented metal/sorbent ratio requires a more frequent mode switch, will thus decrease the COP. An optimized thickness of 450 µm was chosen for further demonstration, which allows the MOF to operate with a working cycle of 30 minutes. In this case, the parasitic sensible loads are one order of magnitude smaller than the latent load.

For comparison, we fabricated other Hex Elements in the same way based on other amphiphilic MOFs and traditional sorbents with an apparent water loading difference within the working range^32,39–44^. Figure 3d shows the thermodynamic and kinetic results of the sorbents. The water sorption cycling speed in MOFs is much more significant than in conventional materials, e.g., the MIL-100(Fe) coating layer adsorbed water 12 times faster than silica gel. The sorption kinetics is important in engineering where the volumetric SCP is a crucial indicator.

### MOF HEx dehumidification performance and energy efficiency

**Figure 4:**
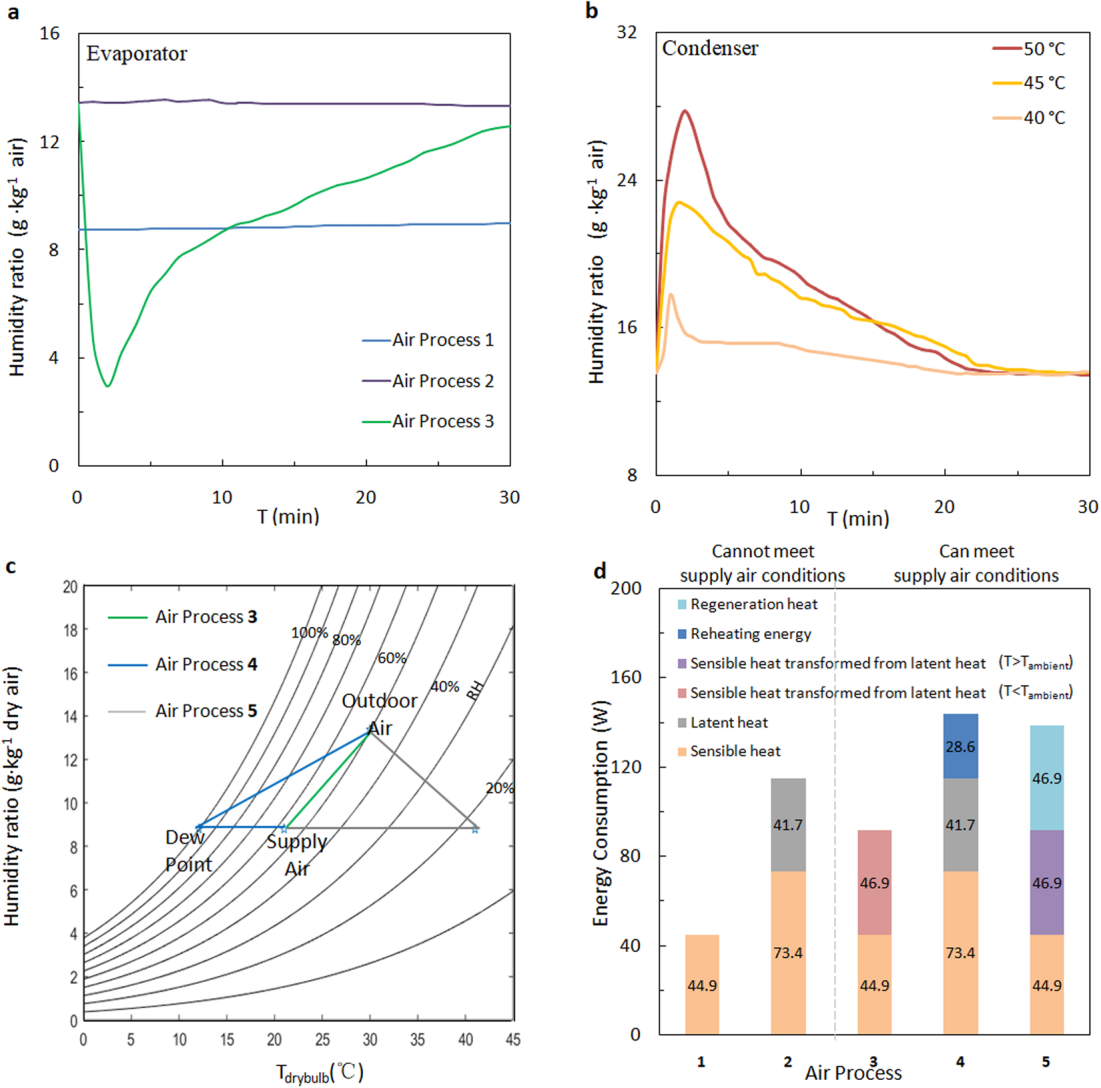
Sorption and energy performance of the proof-of-concept system. (**a**) Supply air humidity ratio from uncoated HEx at evaporation temperature of 19 °C (Air process 1), at 9.5 °C (Air process 2) and MOF HEx at 19 °C (Air process 3). (**b**) Exhaust air humidity ratio from MOF HEx in the desorption mode at 50 °C, 45 °C and 40 °C. (**c**) Psychrometric representation of air process lines for outdoor air of 30 °C, RH 50% and supply air of 21 °C, RH 50%. (**d**) Comparison of energy consumption on sensible and latent heat for the different air processes to reach the same state of supply air.

We validated the concept and demonstrated the benefits in operation of high temperature cooling by investigating a fully functional MOF HEx. The full-scale vapour sorption test consisted of a MOF HEx made of 85.6 g MIL-100(Fe) dip-coated^45^ on the 0.2 mm-thick aluminium fins and 12 mm-diameter copper tubes. The MOF HEx (20 cm × 5 cm × 15 cm) was placed in a thermally insulated test chamber, through which a hot outdoor airflow was passed (30 °C, RH 50%). Two baselines with uncoated heat exchanger were used in comparison with MOF HEx in Fig. 4a: Air process 1, traditional high temperature cooling without dehumidification; Air process 2, traditional vapour-compression cooling with refrigeration-based dehumidification. The supply air of 1 and 2 was 21 °C, RH 87% and 12 °C, RH 95%, respectively. When the evaporation temperature was set to 19 °C, which is slightly above the dew point, MOF HEx in Air process 3 (the proposed system) reduced the humidity ratio of the airflow from 13.6 g·kg^−1^ to an average of 8.5 g·kg^−1^ within the 90% operation time. It corresponded to the dehumidification performance of an uncoated heat exchanger with an evaporation temperature below 11.7 °C. However, we cannot provide the indoor space directly with supply air from either 1 or 2. The extremely humid air from Air process 1 is outside the occupant comfort zone. A standalone dehumidification step is required in the design of a traditional high temperature cooling system^4,46^. On the other hand, the over-cooled air from Air process 2 may cause condensation in the indoor environment, which damages building structures and promote microbial growth^47,48^. The process ends up with an additional air mixing or re-heating step, which increase the complexity of the air circulation system and the sensible heat load.

We have evaluated the energy efficiency of the proposed system (Air process 3). Since the latent load is removed by MOF adsorption under quasi-isothermal conditions, the low efficiency in mass and heat transfer related to moisture condensation was eliminated. The total energy input can be roughly estimated as the sum of the adsorption heat of the MOF coating and the sensible load of the outdoor air plus 30% consumption in fans and other losses, as is general industry practice. It is important to note that the desorption of wet MOFs is completely driven by waste heat from the condenser and no extra energy is used. The corresponding COP_sys_ is 6.6 at a condensation temperature of 50 °C. The COP is very sensitive to the temperature differences between the evaporator, the ambient atmosphere, and the condenser. Each 1 °C reduction of the condenser temperature indicates a saving on energy input by 3%^49^. If the condensation temperature dropped to 45 °C, the COP_sys_ would increase to 7.9. On the other hand, reducing the desorption temperature would result in a longer desorption time and would introduce losses of cyclical water uptake. The performance of MIL-100(Fe) was barely affected, whereas the more hydrophilic MOFs would probably be weakened. For instance, the cyclical water uptake of UiO-66(Zr) and MIL-125(Ti) was reduced by 0.2 and 0.13 kg·kg^−1^ dry mass (−45% and −36%), respectively (SI4). Both the thermodynamic and the kinetic properties of the non-trivial sorbents merit individual studies to find the compromise that how they might be better adapted to different climates and engineering demands.

The COP of the proposed system is one of the best values reported for variable space cooling systems. By increasing the evaporation temperature to above the dew point, the high temperature cooling system has a smaller refrigerant pressure difference between the evaporator and the condenser. In other words, the compressor can reject heat more easily and efficiently. Therefore, the COP of high temperature cooling systems is usually higher than vapour-compression air conditioning. However, the supply air from the traditional high temperature cooling system (Air process 1) is very humid, which cannot meet the required conditions for thermal comfort. An additional dehumidification system is needed. To reach the same state of supply air as Air process 3 (i.e. same temperature, RH and flow rate), we added two more scenarios with commercial technology concepts for comparison: Air process 4, vapour-compression air conditioning with reheating; and Air process 5, stand-alone dehumidification by a desiccant wheel combined with a sensible cooling process (Fig. 4c).

Given the same states of outdoor air (30 °C, 50% RH) and supply air (21 °C, 55% RH), Air process 3 demonstrated significant energy savings compared to the other systems. Figure 4d provides detailed information on the breakdown of the energy consumption of the different air processes to reach the same supply air conditions. The MOF HEx in Air process 3 could save 36.1% energy consumption by avoiding over-cooling and reheating compared to 4 while maintaining a high SCP. In Air process 5, the desiccant wheel transforms the latent heat to sensible heat under isenthalpic conditions, which is less efficient than the isothermal adsorption process in Air process 3. Moreover, the same quantity of additional energy input is required to regenerate the desiccant. The total energy consumption of Air process 5 is 33.8% higher than that of Air process 3.

The cooling power of the MOF HEx in our experiment was 91.8 W. Continuous cooling would require two identical MOF HEx units that operated alternately. The MOF HEx can maintain a cooling power density of up to 82 W·L^−1^ including the removal of latent heat by MOF with 274 W·kg^−1^ of MIL-100(Fe). The high cooling power density makes the proposed system very competitive with most commercial cooling systems^50^.

## Conclusions

We report a new high temperature cooling system that uses amphiphilic MOFs as advanced moisture sorbents with good performance in removing both latent and sensible heat loads simultaneously. The system makes possible an energy-efficient working cycle with a small temperature difference of less than 30 °C between the evaporator and the condenser to exploit the S-shape isotherm of MOFs. The MOF-coated heat exchangers used in the system can remove the cooling load with a power density of 82 W·L^−1^ including latent heat with 274 W·kg^−1^ MIL-100(Fe). In a quasi-isothermal adsorption process, the system eliminates 36.1% of the working load in refrigeration-based dehumidification by a conventional air-conditioner with reheating. The overall COP of the system could be up to 7.9.

We expect that further refinement of the sorbent processing and shaping could optimise the working cycles and enhance the efficiency that can be achieved in different climates. The high COP, high specific power and the minimal modification of existing air conditioning systems that is required makes this new technology suitable for adoption in a broad range of applications and provides a pathway towards highly energy-efficient space cooling.

## Methods

### Syntheses of MOFs

Large-scale MIL-100(Fe) or Fe_3_O(H_2_O)_2_OH(BTC)_2_ was prepared as in^29^. The additive free reaction comprising iron nitrate, trimesic acid and water as a sole solvent takes place at low temperature (60 °C) under stirring. The activation conditions consist in washing the solid with water at room temperature. The synthesis procedures of other MOFs can be found in SI1.

### Coating on heat exchanger element with MOFs powders

Aluminium sheets (4 cm × 4 cm × 0.03 cm) were cleaned in acetone, and then immersed in 0.3 mol·L^−1^ CH_3_COOH and 1 mol·L^−1^ NaOH for 1 min, respectively, before being rinsed with deionized water. A slurry was obtained by dispersing 1.00 g MOF powder in 3.03 g of deionized water with vigorous stirring for 10 min followed by supersonic treatment for 20 min. The binder silicate sol (0.62 mL, 50 wt%) was added to the suspension with continuous stirring. The aluminium sheets were coated manually with the suspension on a single side of the aluminium sheet, using a pipette. The coated sheets were dried at 50 °C for 1 h, and cured at 150 °C for 2 h.

### Coating on full-scale heat exchanger with MIL-100 powders

The MIL-100(Fe) suspension was prepared by using the above procedure (300 g MIL-100(Fe), 600 g deionized water and 102 mL silicate sol). The heat exchanger was dip-coated by immersion in the suspension, dried and cured following the heating procedure described above. Extra slurry on the edges was removed mechanically. The coating was repeated twice to obtain the desired thickness, resulting in 85.6 g of dried coating sorbent.

### Water sorption characterization of the MOF HEx Element

The MOF HEx Element was placed in a small climate chamber (60 cm × 40 cm × 100 cm). A high precision humidity generator (SETARAM Instrumentation, Wetsys) provided the inlet air with controlled temperature and relative humidity. The temperature regulator for the MOF HEx Elements comprised a Peltier cooler (TE Technology, TEC1-12703, 4 cm × 4 cm × 0.4 cm) with two metal heat buffers on each side (Aluminium plates, 4 cm × 4 cm × 3 cm), bonded with thermally resistant grease. A small space (5 mm × 5 mm × 1 mm) was milled out of the aluminium heat buffer at the corner on the MOF HEx side to insert the thermocouple temperature sensors (Omega Type K HFS-4) for the *in-situ* thermal measurement. The TEC was linked to an amplifier (HH-electronic) to guarantee a low power output (the exothermic adsorption power was about 0.1 to 1 W). A small fan (0.2 W) was installed below the heat sink to assist in air mixing. The climate chamber is large enough to be able to maintain an isobaric environment.

The water sorption characterization consisted of two tests: low-temperature adsorption and high-temperature desorption. One temperature-humidity sensor was placed near the HEx Element to measure ambient conditions, and two thermocouples were inserted into the aluminium heat buffers to measure the temperature of the coating layer and heat sink during adsorption. Before the sorption characterization, the MOF HEx Element was dried at 120 °C. After cooled down, the MOF Hex Element was attached to aluminium heat buffer and the TEC. The entire device was sealed and transported to the small climate chamber in an airtight container. The container was removed and the TEC then started to cool the HEx Element. The temperature for cooling was set above the dew point according to the operating intervals in Fig. 2b, i.e., 20 °C. During the desorption test, the current passing through the TEC was reversed, and the coated HEx Element then became the heat sink. The temperature of the HEx Element was raised to 40 °C, 45 °C or 50 °C to represent the condenser. The tests were repeated many times to measure the stabilized cyclical sorption performance.

### Air process test for full-scale MOF-coated heat exchanger

The full-scale coating test was carried out on a water/air finned-tube heat exchanger with aluminium fins 0.2 mm thick pressed onto copper tubes of 7.5 mm diameter. Fin spacing 1.7 mm, fin volume 120 mm × 25 mm × 125 mm = 0.37 L, total dimension 150 mm × 25 mm × 150 mm = 0.56 L, the total mass 225 g. The HEx was placed in a thermally insulated sample box with the same volume, connected to an airflow with controlled humidity and temperature. Temperature and humidity sensors were installed both upstream and downstream of the HEx in the air duct. The water in a thermostatic bath was circulated through the copper tubes to provide accurate temperature control of the HEx. The tests followed the same temperature profiles of the HEx Element sorption characterization. The incoming airflow to the MOF HEx was 200 L·min^−1^.

## Electronic supplementary material

Supplementary Information

## Data Availability

The data that support the findings of this study are present in the paper and the Supplementary Information. Additional information is available from the authors upon reasonable request.
