# Supplementary material for: Metal-Organic Frameworks as advanced moisture sorbents for energy-efficient high temperature cooling
Source: Sci Rep. 2018 Oct 16;8:15284. doi: 10.1038/s41598-018-33704-4 (PMC6191459; doi:10.1038/s41598-018-33704-4)
Supplement: Supplementary file 1 — Supplementary Information [file 41598_2018_33704_MOESM1_ESM.pdf]

Metal-Organic Frameworks as advanced moisture sorbents for energy-efficient high temperature cooling

Supplementary Information

Shuqing Cui[1, 3], Menghao Qin[1*], Afsaneh Marandi[2], Victoria Steggles[2],Sujing Wang[2],

Xiaoxiao Feng[1], Farid Nouar[2], Christian Serre[2]

[1]Department of Civil Engineering, Technical University of Denmark, Lyngby 2800, Denmark.

[2]Institut des Matériaux Poreux de Paris, FRE 2000 CNRS, Ecole Normale Supérieure, Ecole Supérieure de Physique et de Chimie Industrielles de Paris, PSL Research University, 75005 Paris, France.

[3]Elektron Gri, 75005 Paris, France.

*Corresponding author's email: menqin@byg.dtu.dk

SI1. Synthesis and characterization of MOFs

All chemicals were purchased from the market (Sigma Aldrich) without further purification.

Mil-125(Ti) or $Ti_8O_8(OH)_4(BDC)_6$: Terephthalic acid (91.5 mmol, 15.2 g) and titanium isoproproxide $Ti(OiPr)_4$ (62.1 mmol, 18.4 ml) were mixed in a solution of DMF (320 mL), methanol (80 mL) and water (1 mL). The mixture was then heated to reflux for 72 hours. A white precipitate was recovered by filtration, washed with DMF (1.8 L) for 16 hours and methanol (1.5L) for 16 hours. The solid was finally recovered via filtration and heated to 200°C for 16 hours.

MOF-808(Zr) or $Zr_6O_4(OH)_4(BTC)_2$: Trimesic acid (BTC, 1.49 g) was transferred into a thick-glass jar followed by adding DMF (128 mL). While stirring at room temperature, $ZrOCl_2\cdot8H_2O$ (2.16 g) was added to the reaction solution followed by adding formic acid (133 mL). The resulting solution was stirred at room temperature for 20 minutes. Then the jar was sealed well and heated in oven to 110 °C for 48 hours. After cooled to room temperature, the crude product was collected with centrifugation while the product should be kept wet at this stage. The product was refluxed in DMF and acetone separately for activation, and then dried in air after filtration.

Basolite A520 or AlOH(Fum): A solution of fumaric acid (4.3 mol, 522 g), NaOH (13.5 mol, 540 g) and water (10 L) was prepared, stirred and heated to 60°C (Solution 1). A second solution of $Al_2(SO_4)_3\cdot18H2O$ (4.3 mol, 1476 g) in water (3.5 L), stirred and heated to 60°C (Solution 2). Solution 2 was then slowly added to solution 1 resulting in the precipitation of a white solid. The mixture was kept at 60°C for few minutes and the solid was recovered by filtration and washed in water (15 L) at 60°C under stirring for about 3 hours. Finally, a white powder was recovered via filtration and dried in a vacuum oven for 1 day at 130°C.

UiO-66(Zr) or $Zr_6O_4(OH)_4(BDC)_6$: Dimethyl terephthalate (16 mmol, 3.1 g) and $ZrCl_4$ (16 mmol, 3.7 g) were mixed in propan-2-ol (16 mL) with HCl (64 mmol, 5.351 mL) . The mixture was heated under reflux for 70 hours. White solid was recovered by filtration after cooled down. The powder was washed twice in ethanol at 50 °C (1g in 50 mL).

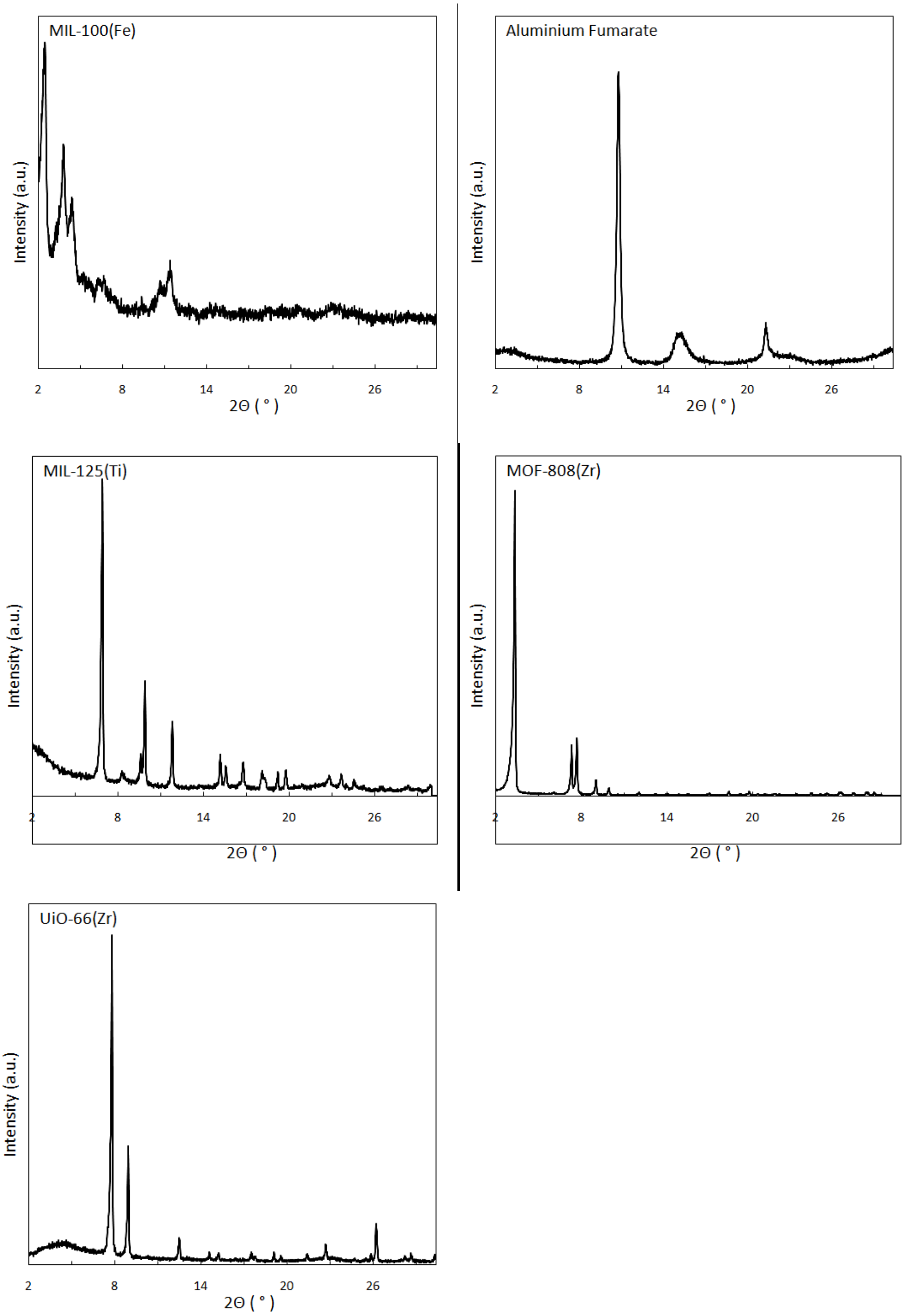

Fig. S1. X-ray powder diffraction (λ = 1.540598 Å) for MIL-100(Fe), Basolite A520 (Aluminium Fumarate), MIL-125(Ti), MOF-808(Zr), and UiO-66(Zr)

Adsorption isotherms (N_2)

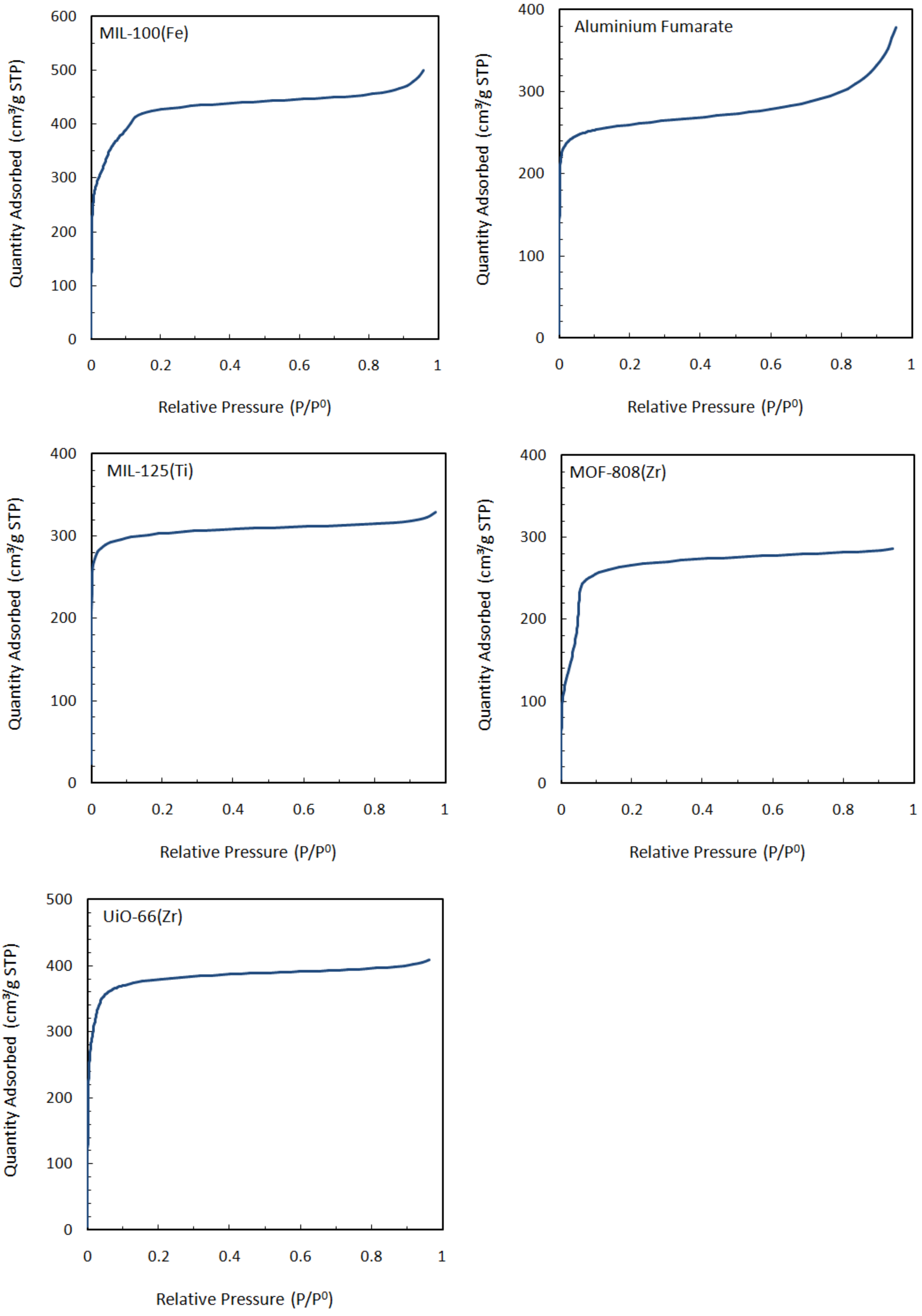

Fig. S2a. Nitrogen gas sorption isotherms of the MOFs. Mil-100(Fe), Basolite A520(Fumarate Aluminum), UiO-66(Zr), MOF-808(Zr) and MIL-125(Ti)

Adsorption isotherms (H_2O)

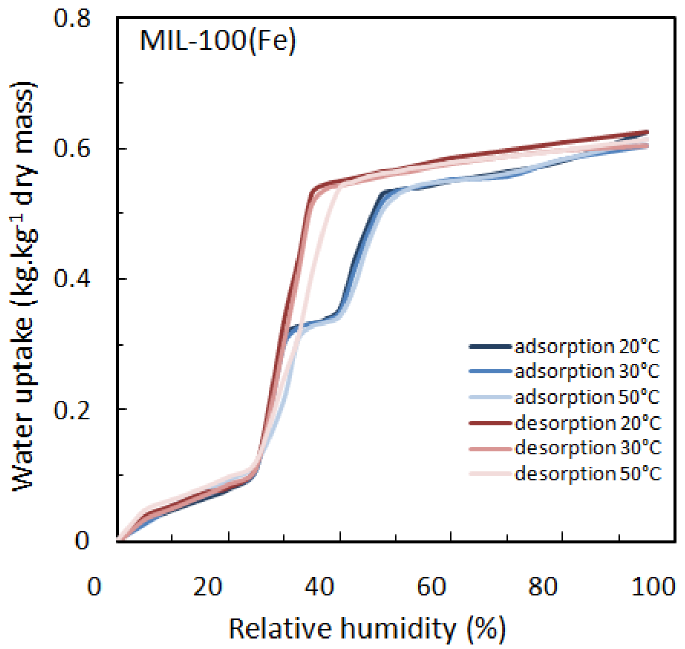

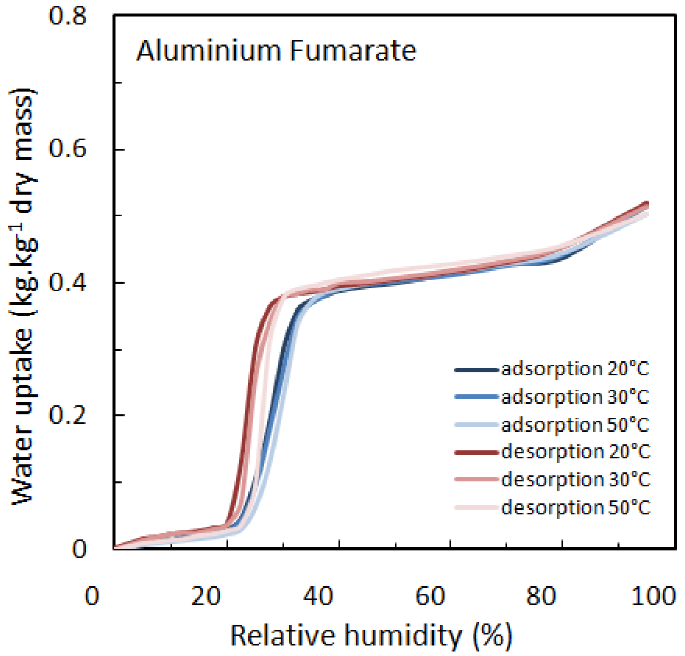

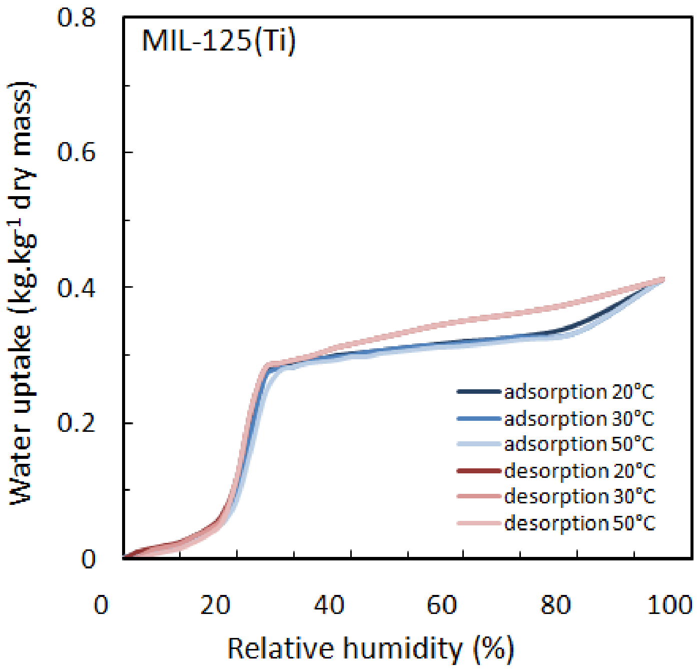
MIL-125(Ti)
0.8
0.6
0.4
0.2
0
Water uptake (kg.kg-1 dry mass)
0
20
40
60
80
100
Relative humidity (%)
adsorption 20°C
adsorption 30°C
adsorption 50°C
desorption 20°C
desorption 30°C
desorption 50°C

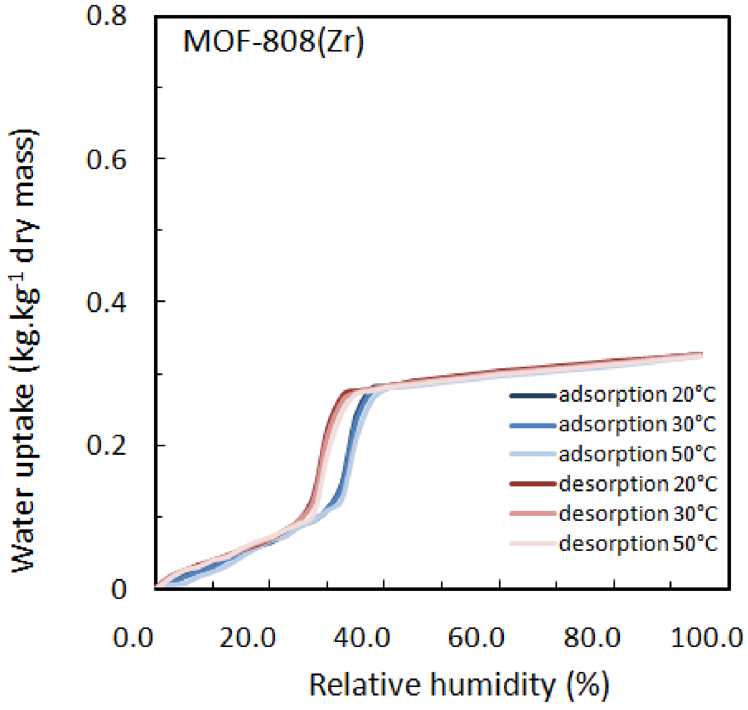
MOF-808(Zr)
0.8
0.6
0.4
0.2
0
Water uptake (kg.kg-1 dry mass)
0.0
20.0
40.0
60.0
80.0
100.0
Relative humidity (%)
adsorption 20°C
adsorption 30°C
adsorption 50°C
desorption 20°C
desorption 30°C
desorption 50°C

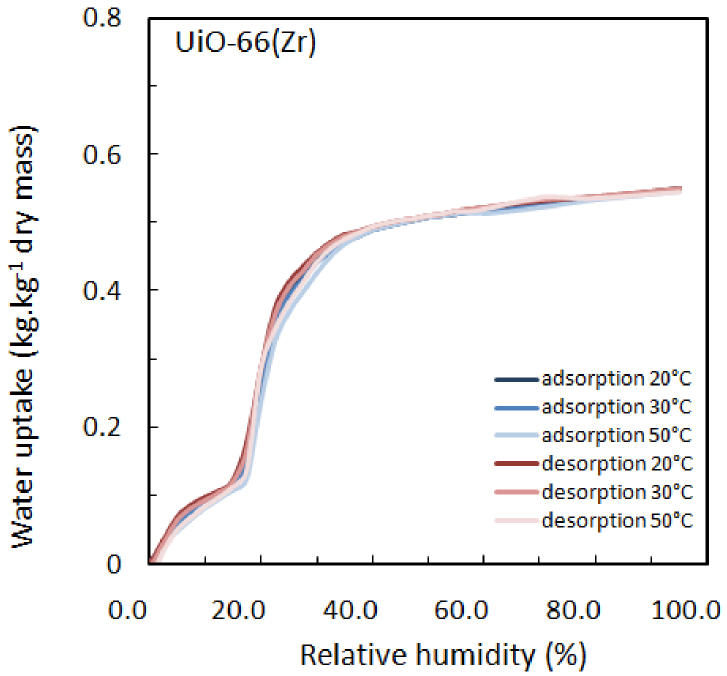
UiO-66(Zr)
0.8
0.6
0.4
0.2
0
0.0
20.0
40.0
60.0
80.0
100.0
Water uptake (kg.kg-1 dry mass)
Relative humidity (%)
adsorption 20°C
adsorption 30°C
adsorption 50°C
desorption 20°C
desorption 30°C
desorption 50°C

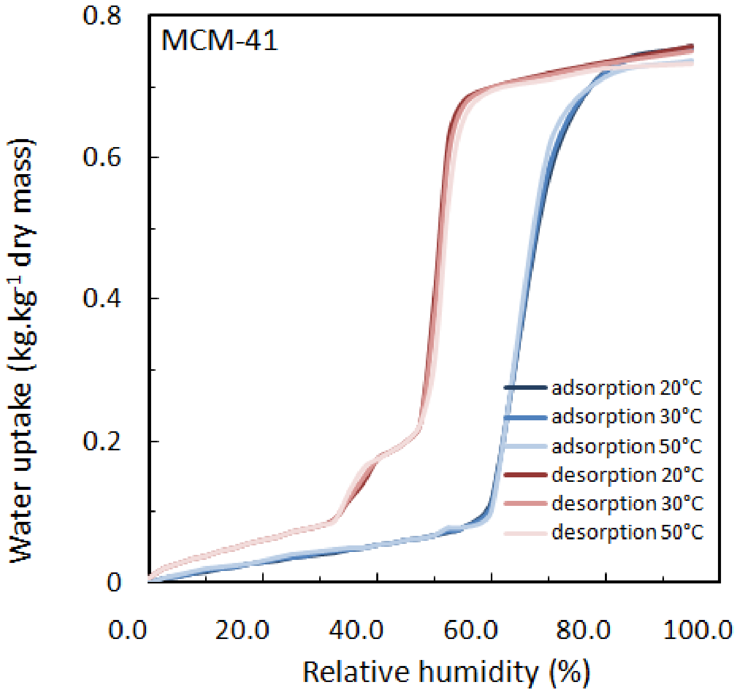
MCM-41
0.8
0.6
0.4
0.2
0
0.0
20.0
40.0
60.0
80.0
100.0
Water uptake (kg.kg-1 dry mass)
Relative humidity (%)
adsorption 20°C
adsorption 30°C
adsorption 50°C
desorption 20°C
desorption 30°C
desorption 50°C

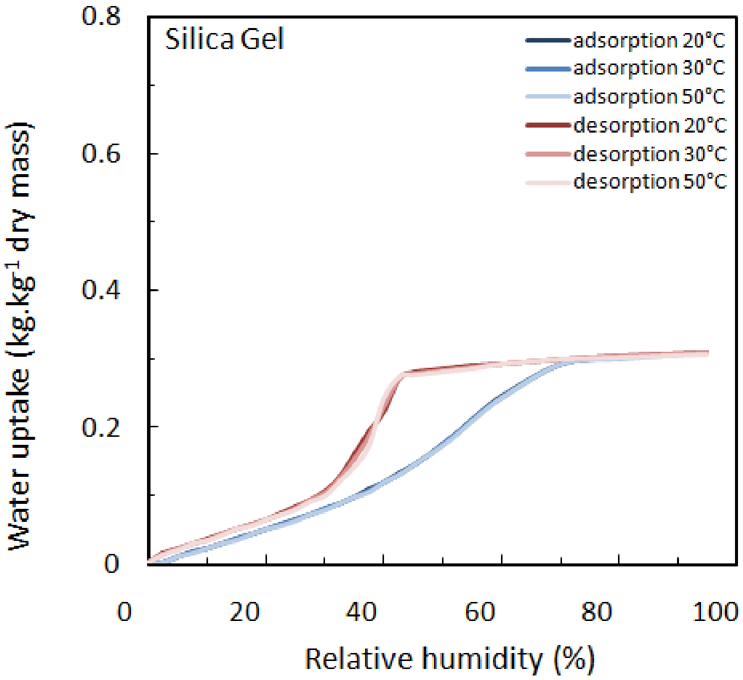

Fig. S2b. Water sorption isotherms of the sorbents. MIL-100(Fe), Basolite A520 (Fumarate Aluminum), UiO-66(Zr), MOF-808(Zr), MIL-125(Ti), MCM-41 and Silica Gel. The characterizations were performed by a TG-DVS instrument (Surface Measurement Systems DVS Adventure).

MOF Heat Exchanger

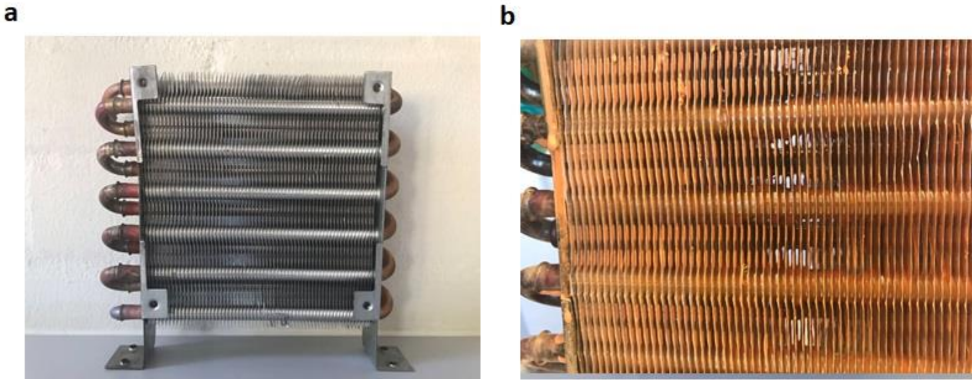

Fig. S3. Heat Exchanger coated with Mil-100(Fe) powder. a Uncoated heat exchanger. **b** Heat exchanger after 2 coating processes. Details of the fins.

Coating effects

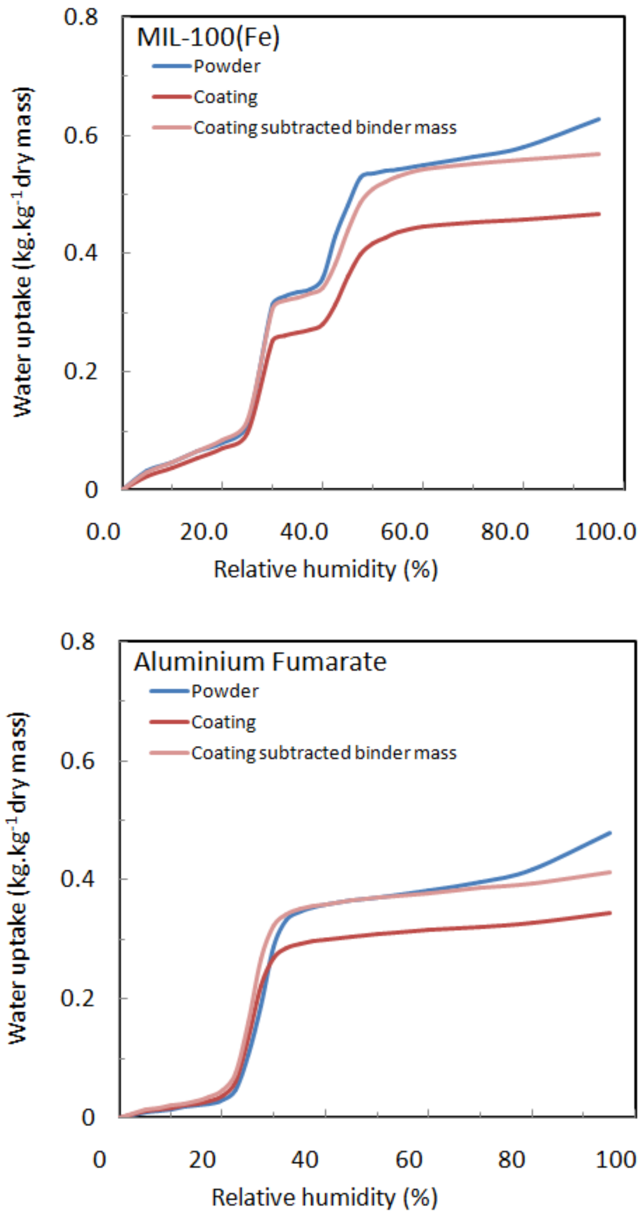

Fig. S4. Water adsorption isotherms of MIL-100(Fe) and Basolite A520 (Aluminium Fumarate) powder, coating samples and and coating samples with subtracted binder mass.

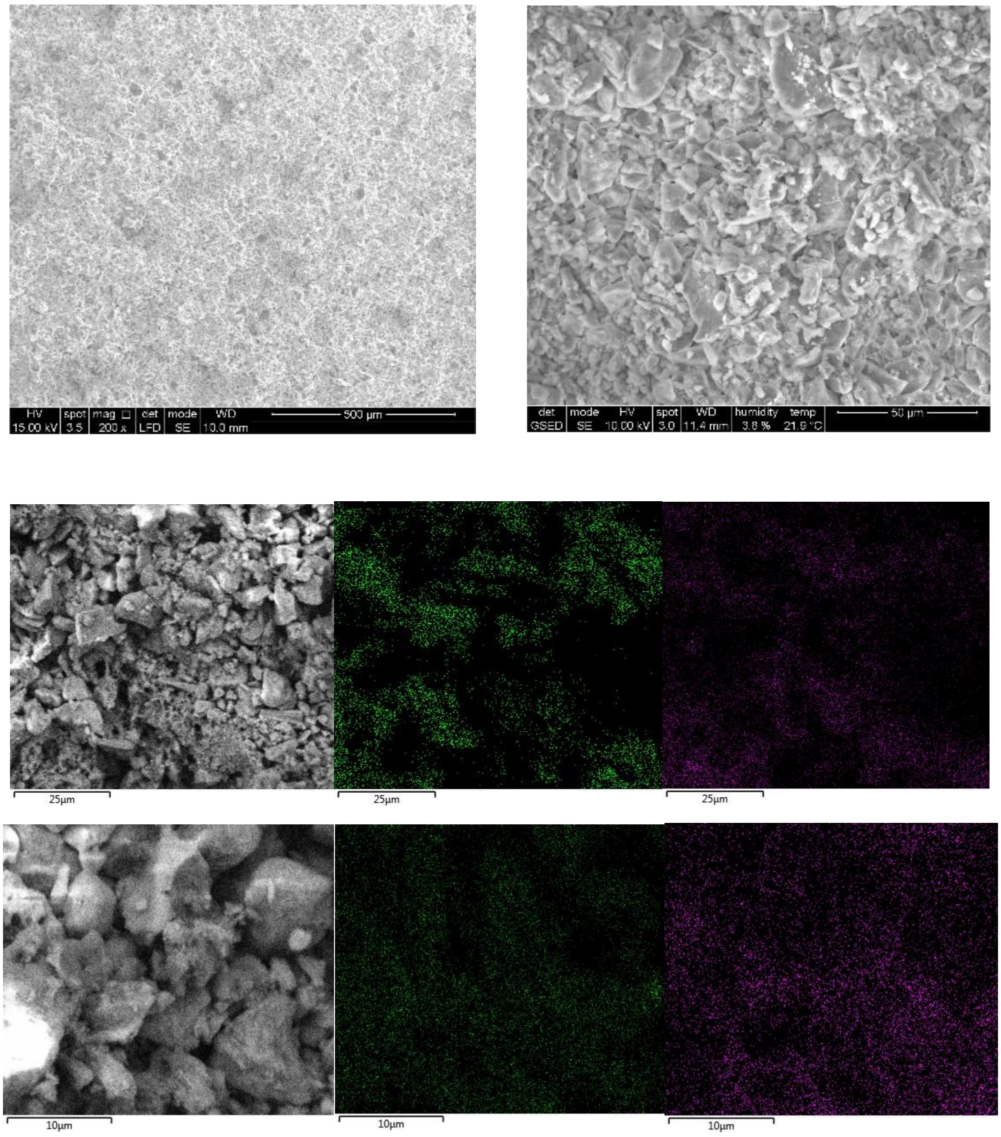

Fig. S5. SEM images of MIL-100(Fe) coating sample. The characterizations were performed by an FEI Quanta 200 ESEM FEG microscope equipped with an energy-dispersive X-ray (EDS) spectrometer. The binder reinforced the mechanical strength of the MOF, with Si element well dispersed in the resulting mixture.

SI2. Stability of MOF coating layer

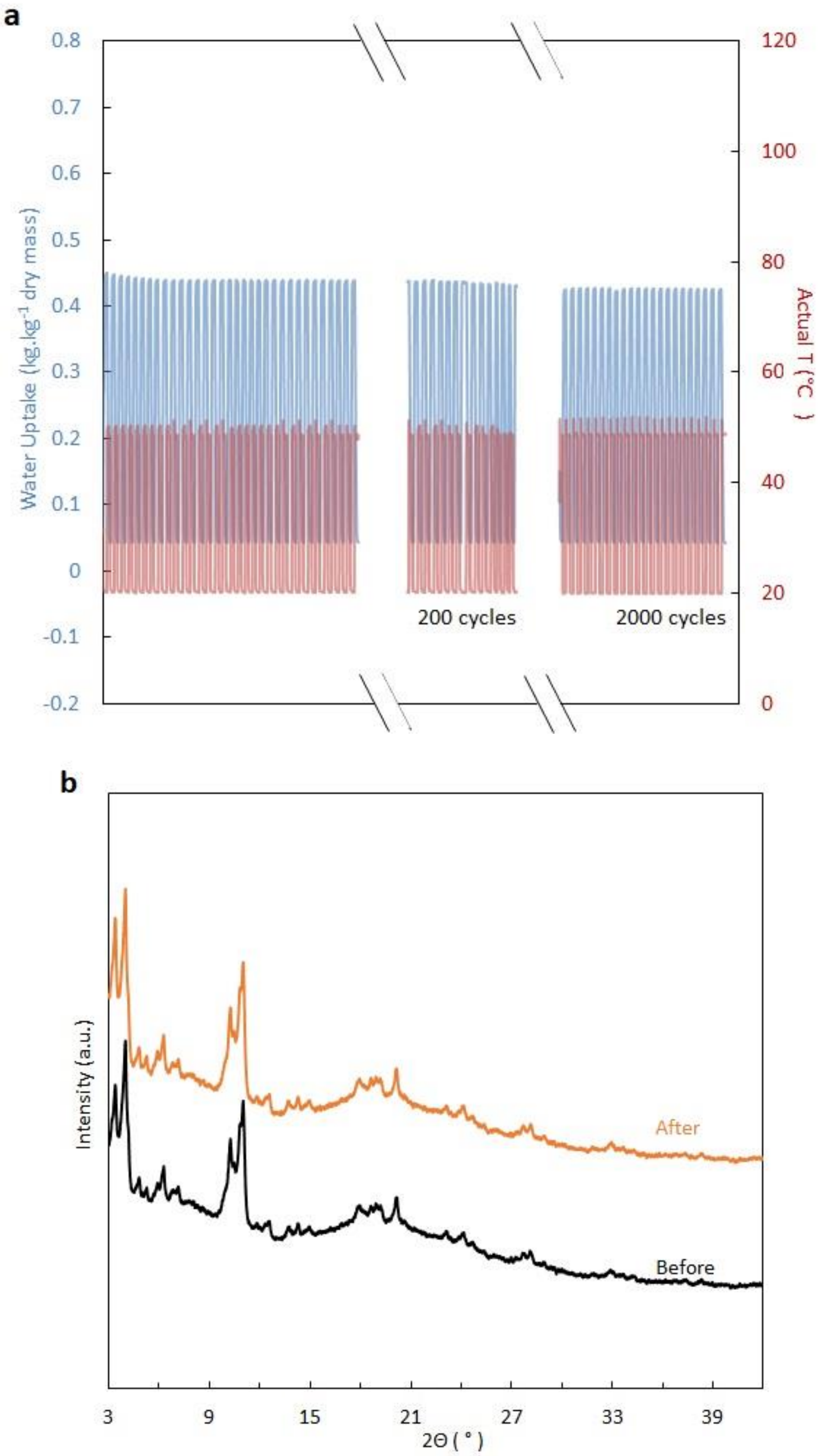

Fig. S6. Hydrothermal stability MOF coating layer. Adsorption uptake of MIL-100(Fe) coating sample was tested over three months for 2000 cycles. **a** The water uptake capacity, a loss of 4.5 wt% in the maximum uptake was observed, and the minimum uptake remained quite stable. **b** X-ray powder diffraction (λ = 1.5406 Å) for the sample before and after the cycle tests, indicating the crystalline stability of coated MIL-100(Fe).

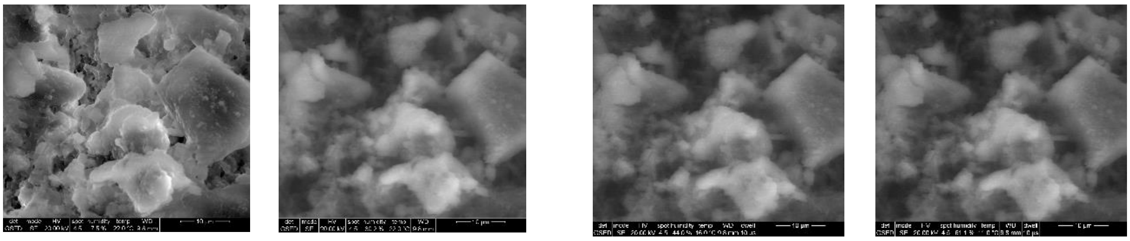

RH7.5% RH30% RH45% RH60%

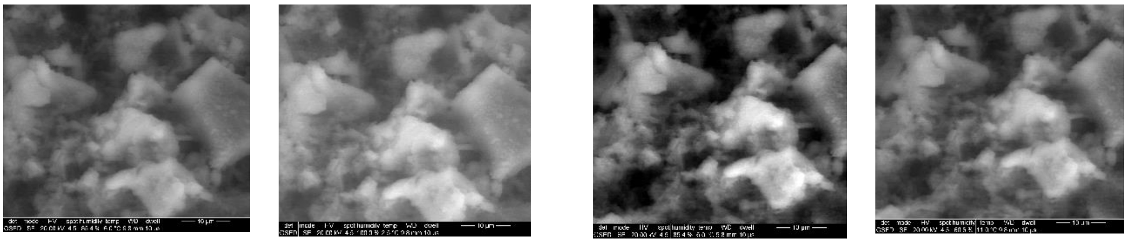

RH85% RH100% RH85% RH60%

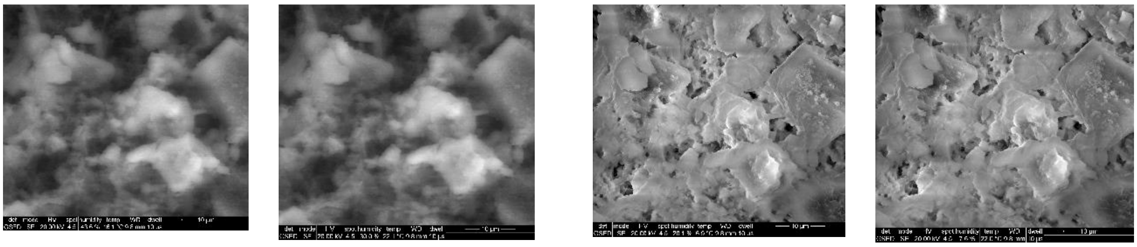

RH45% RH30% RH20% RH7.5%

Fig. S7. ESEM images of MIL-100(Fe) coating sample. Humidity was allowed to vary from 0% to 100% and the sample was exposed for long enough in each state to reach equilibrium. No visible change of the surface was observed.

SI3. Experimental set-up

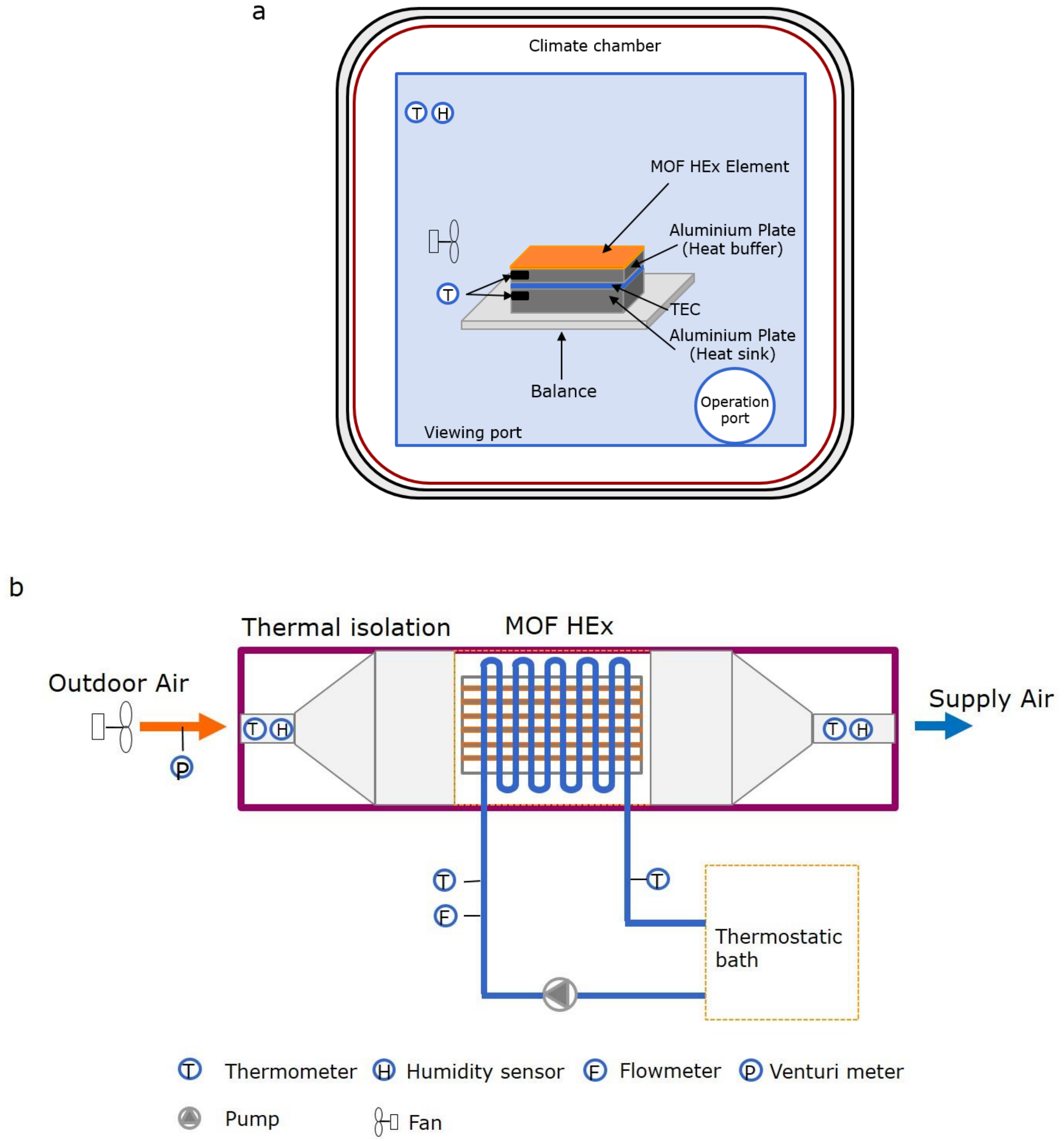

Fig S8. Schematic presentation of experimental set-up. a MOF HEx Element water sorption characeterization. **b** MOF HEx air process.

SI4. Effect of condensation temperature on water uptake capacity

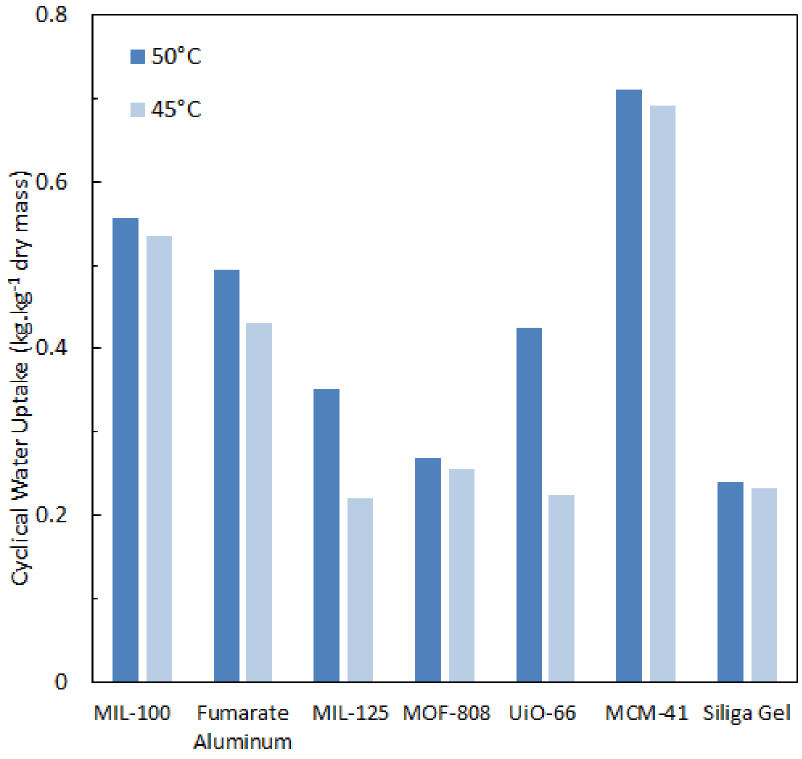

Fig.S9 Water uptake capacity of MOFs at two desorption temperatures. The inlet air was30°C and RH 50% , the evaporation temperature was 20°C and condensation temperature was 45°C /50°C

SI5. Operation range of high temperature cooling with no condensation

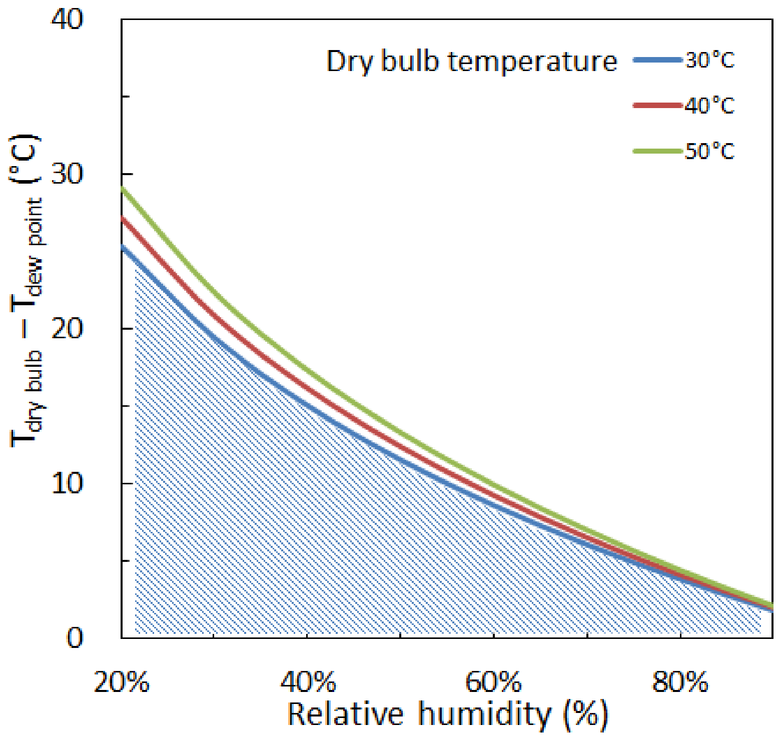

Fig.S10 Operation temperature ranges of cooling with no condensation, where the evaporation temperature is above dew point. The more humid the ambient air, the smaller the temperature difference to the cold source can be achieved without condensation.
